# Supplementary material for: Analysis of 1321 Eubacterium rectale genomes from metagenomes uncovers complex phylogeographic population structure and subspecies functional adaptations
Source: Genome Biol. 2020 Jun 8;21:138. doi: 10.1186/s13059-020-02042-y (PMC7278147; doi:10.1186/s13059-020-02042-y)
Supplement: Supplementary file 1 — Additional file 1. Supplementary Figures. [file 13059_2020_2042_MOESM1_ESM.pdf]

Supplementary Figures

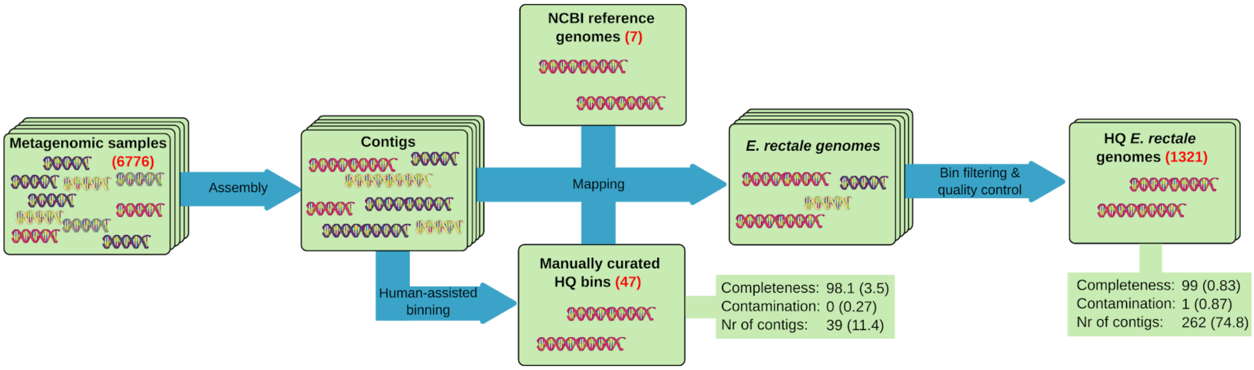

Fig S1: An integrated, reference-based workflow for genome reconstruction from metagenomes. Numbers in red parentheses correspond to the set size. Numbers for completeness, contamination and the number of contigs correspond to the mean and standard deviation (in parenthesis).

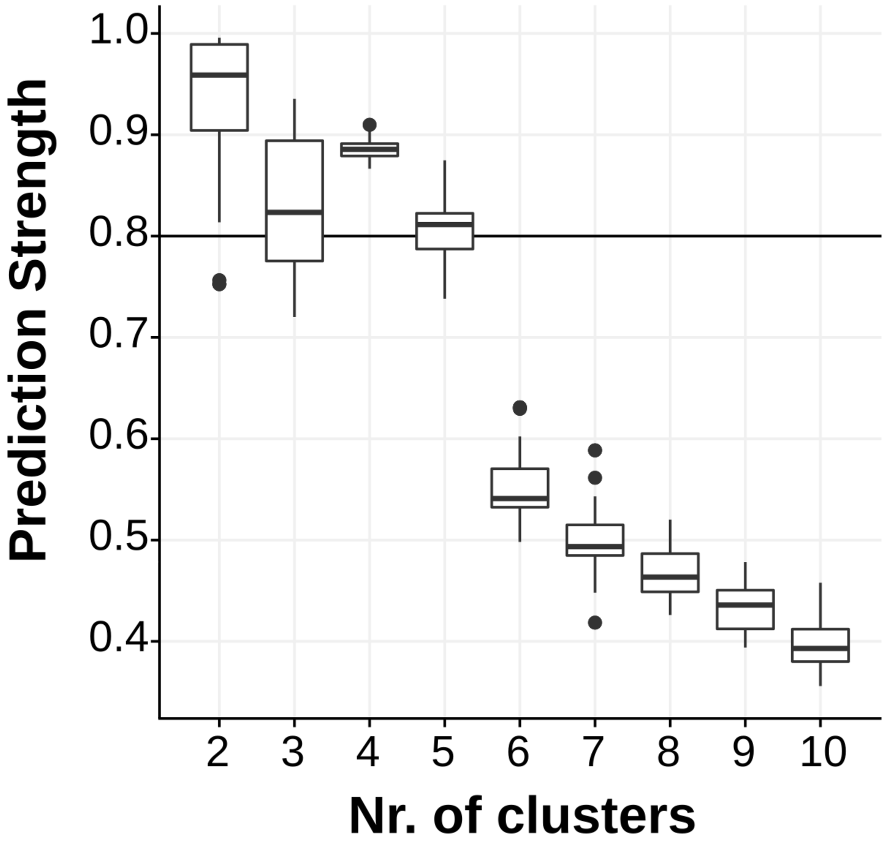

Fig S2: Prediction Strength values for varying numbers of clusters obtained by Partitioning Around Medoids (PAM) clustering (**Methods**). The horizontal line corresponds to a Prediction Strength value of 0.8, suggested by Tibshirani et al. as a cutoff for adequate clustering strength [71].

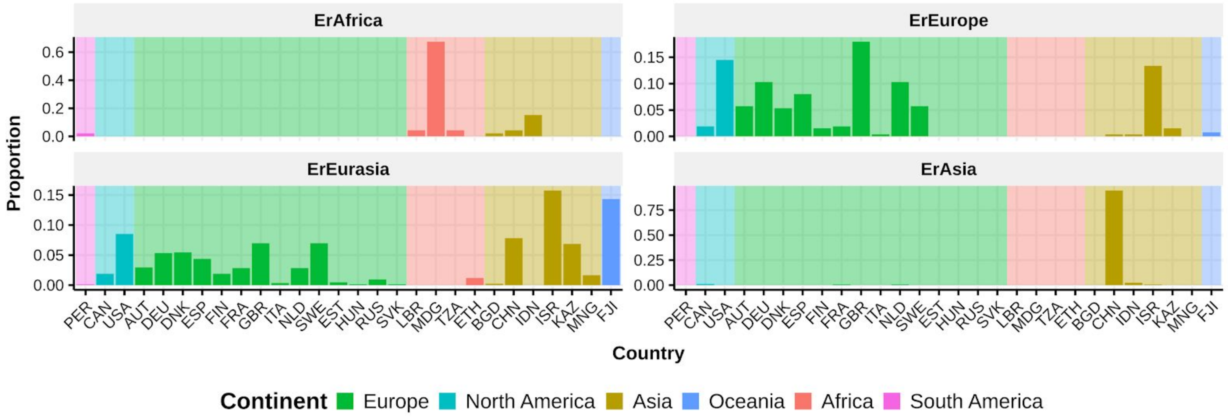

Fig S3: Proportions of *E. rectale* subspecies over countries and continents. Proportions sum up to 1 for each subspecies.

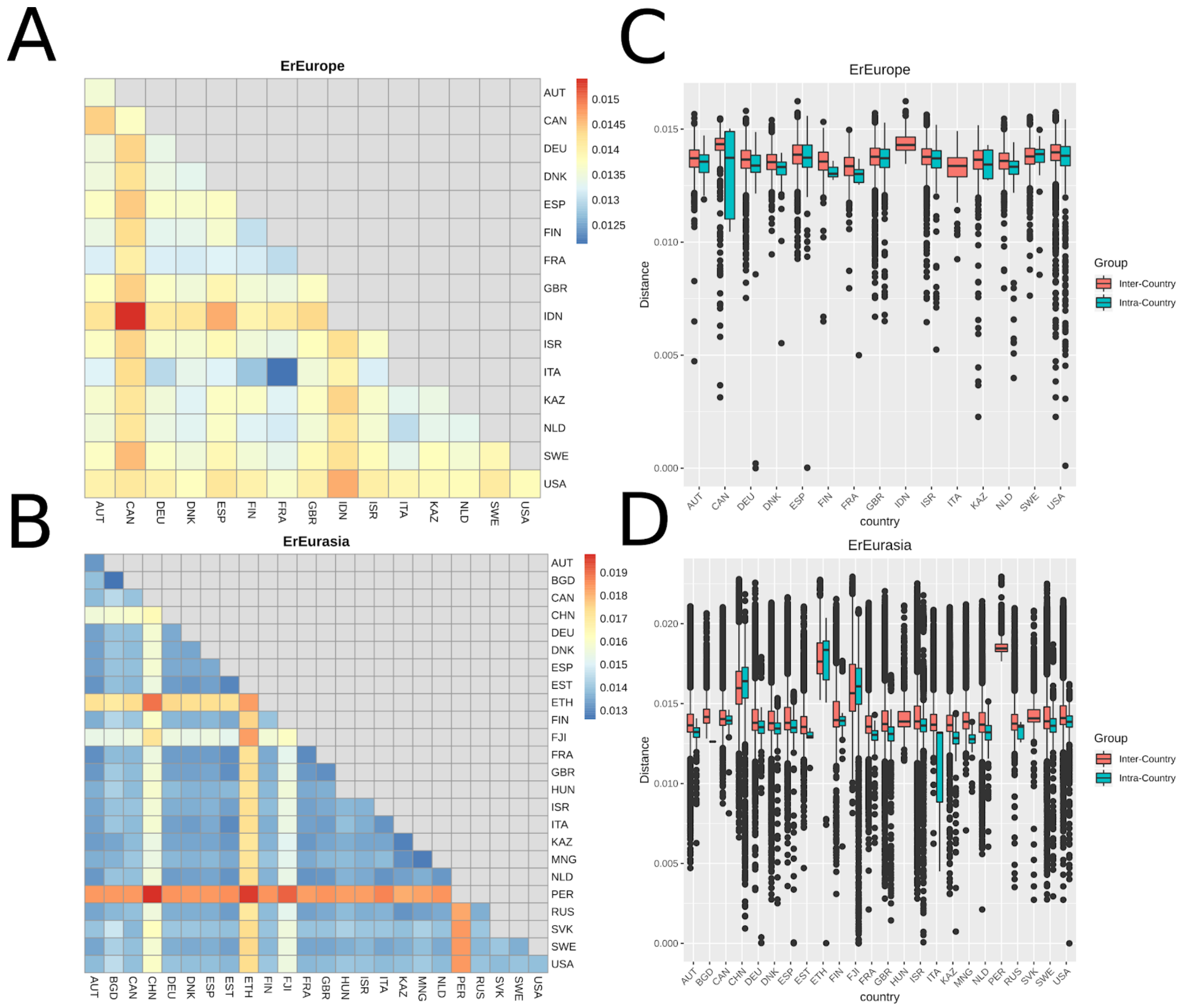

Fig S4: (**A**, **B**) Heatmaps of median pairwise genetic distances between countries considering ErEurope (**A**) or ErEurasia (**B**) individually. (**C**, **D**) Boxplots of within- and between country genetic distances considering ErEurope (**C**) and ErEurasia (**D**) individually.

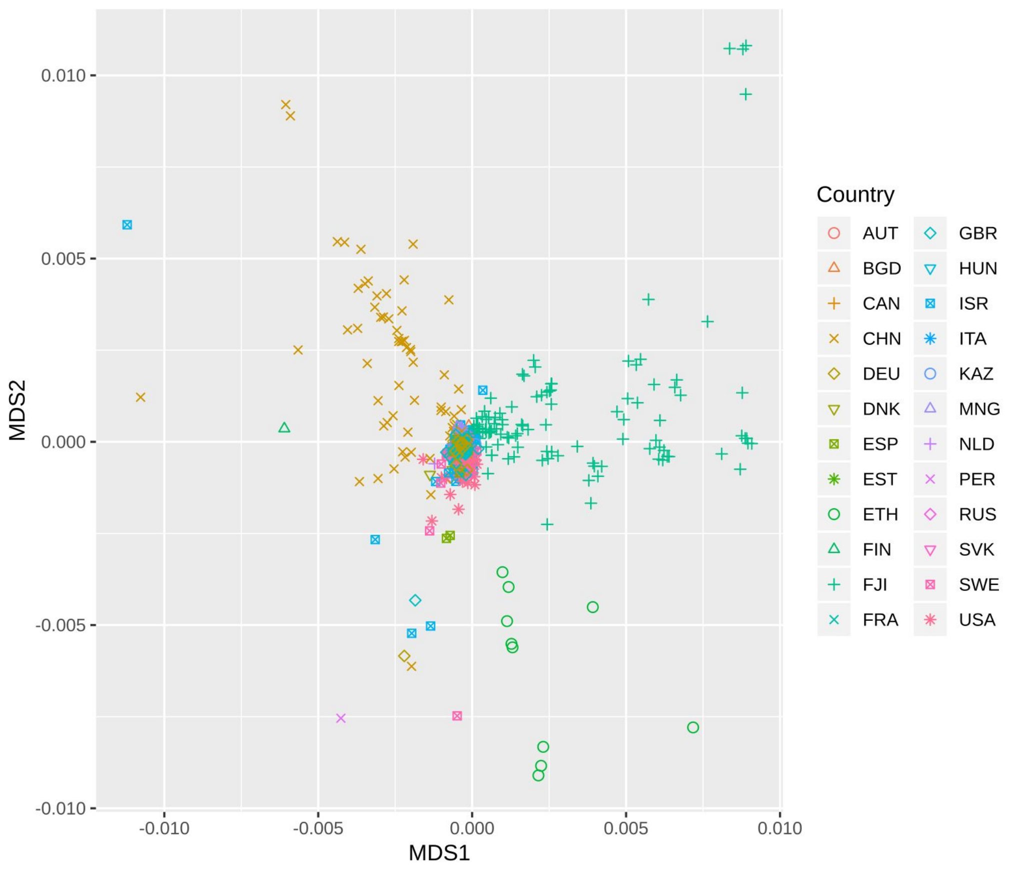

Fig S5: nMDS plot of ErEurasia. See **Additional File 1: Fig. S6** for comparison.

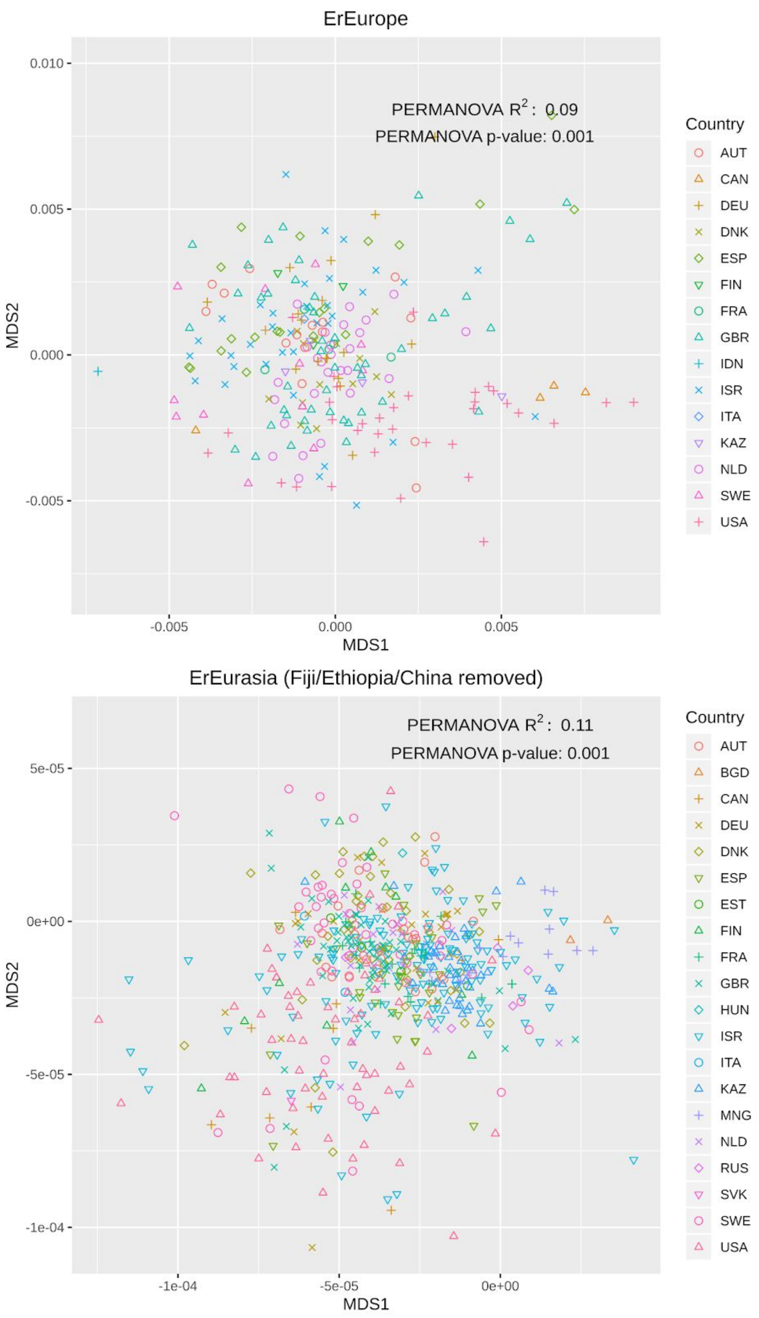

Fig S6: nMDS plots of ErEurope and ErEurasia based on pairwise genetic distances. PERMANOVA was calculated using Country membership. For ErEurasia, we removed Fijian, Chinese, Ethiopian and Peruvian strains for PERMANOVA calculation and from the ordination plot. 24 outlier samples were further removed to facilitate visualization; most of these came from the USA or Israel. See **Additional File 1: Fig. S5** for comparison.

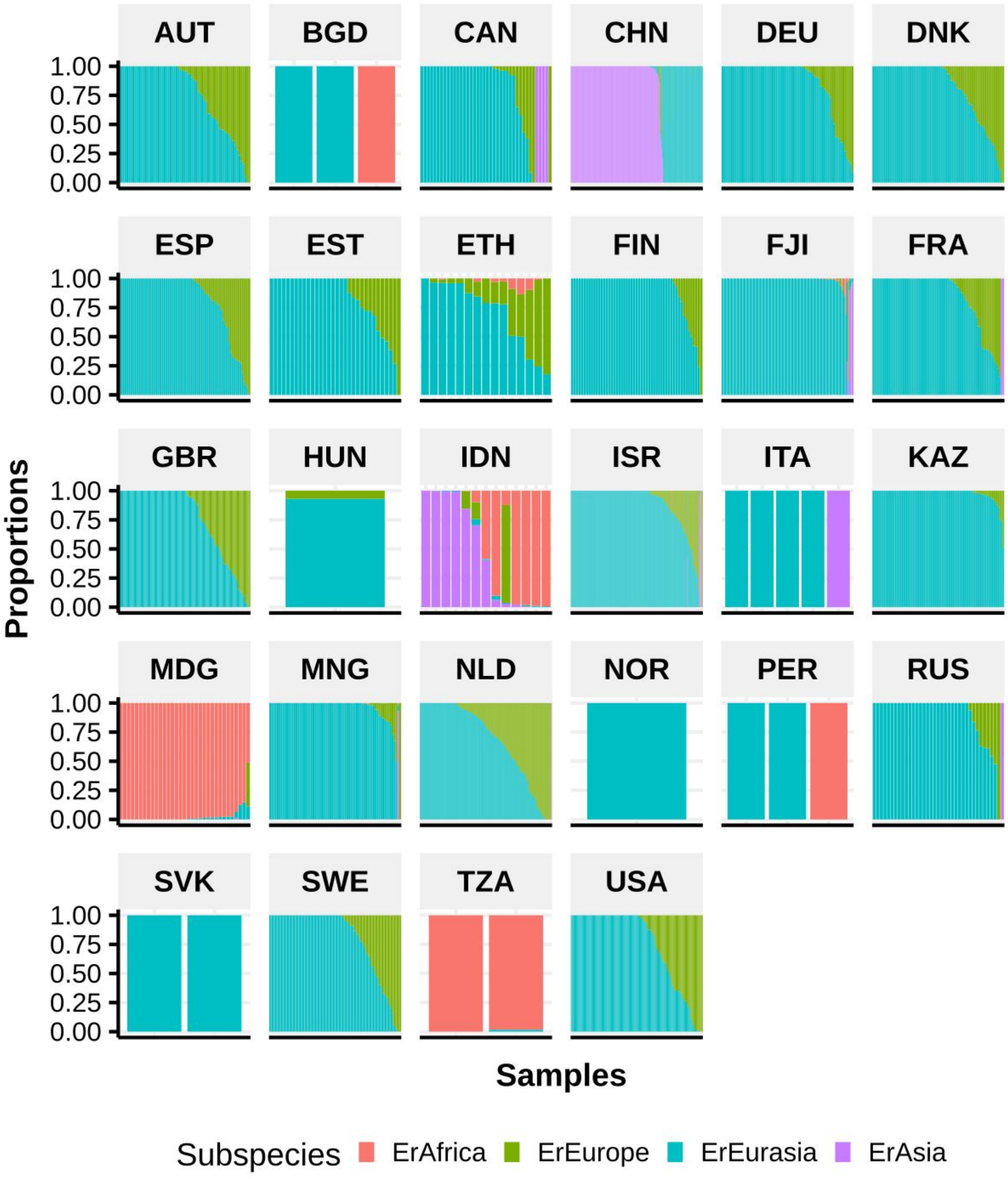

Fig S7: Barplots of subspecies relative abundances over all metagenomic samples that had sufficient coverage over subspecies-specific SNVs (**Methods**).

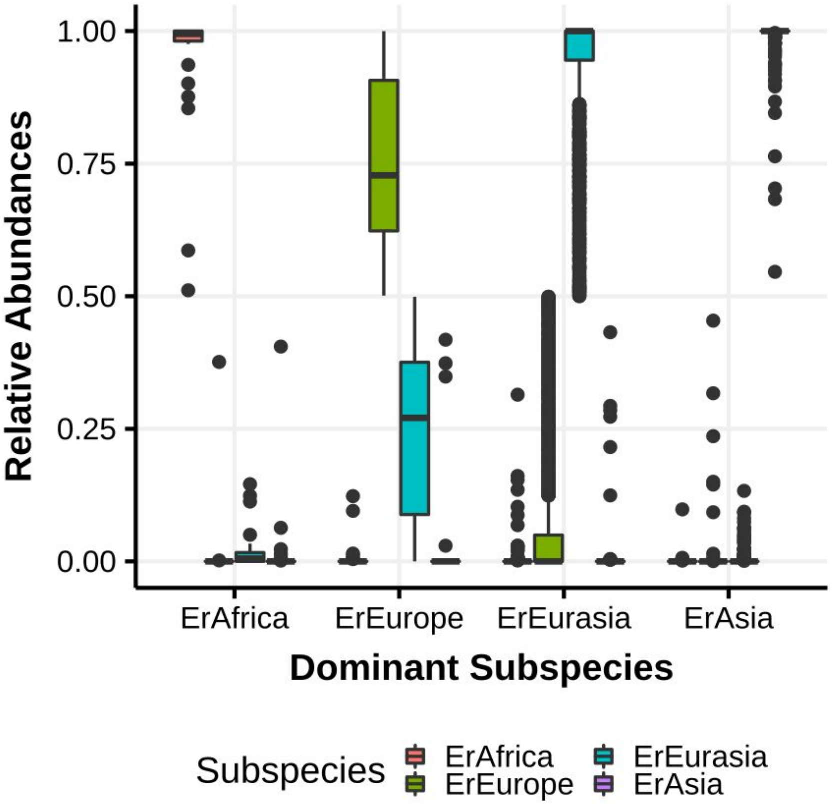

Fig S8: Boxplots of subspecies relative abundances over all metagenomic samples that had sufficient coverage over subspecies-specific SNVs (**Methods**).

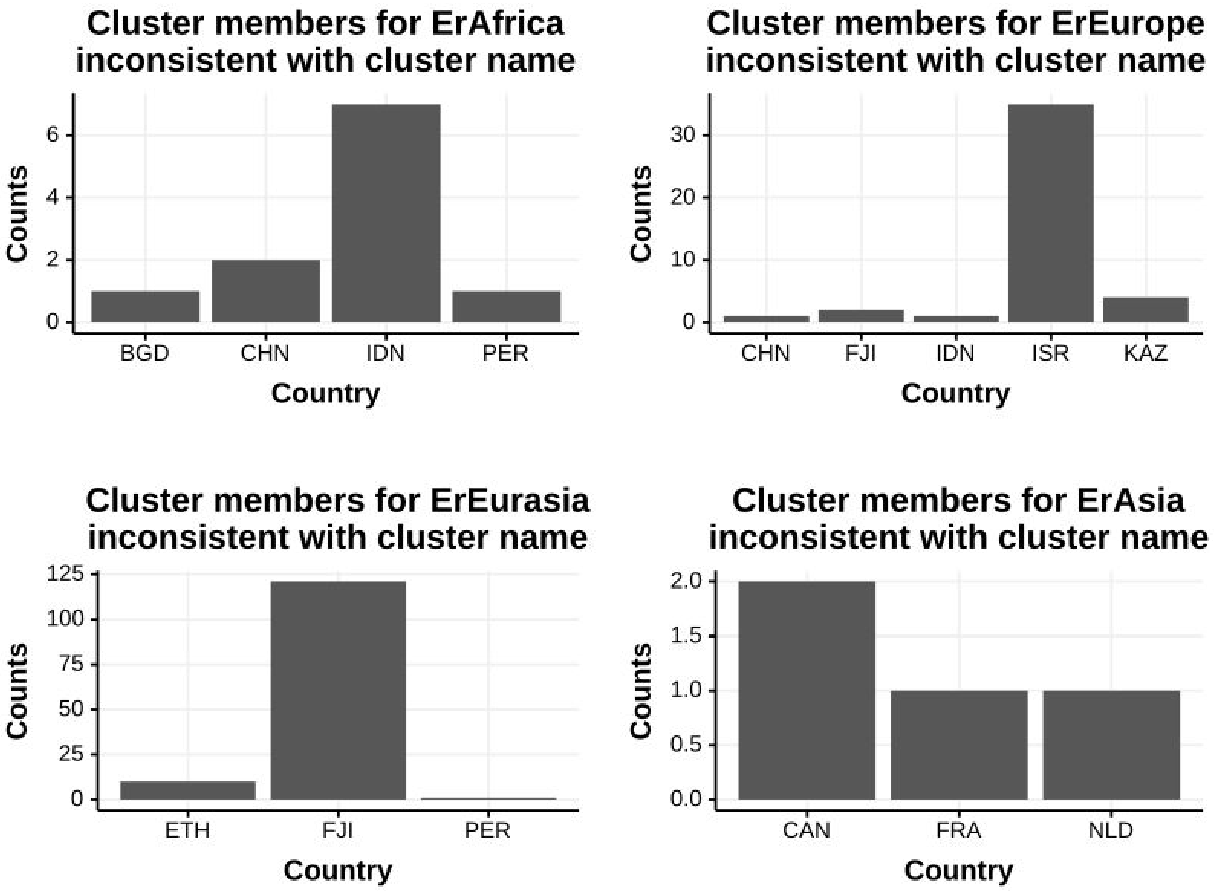

Fig S9: Barplots showing the country of origin of strains showing inconsistent membership (with respect to the subspecies name). For ErEurope and ErEurasia, strains coming from North America are not considered inconsistent.

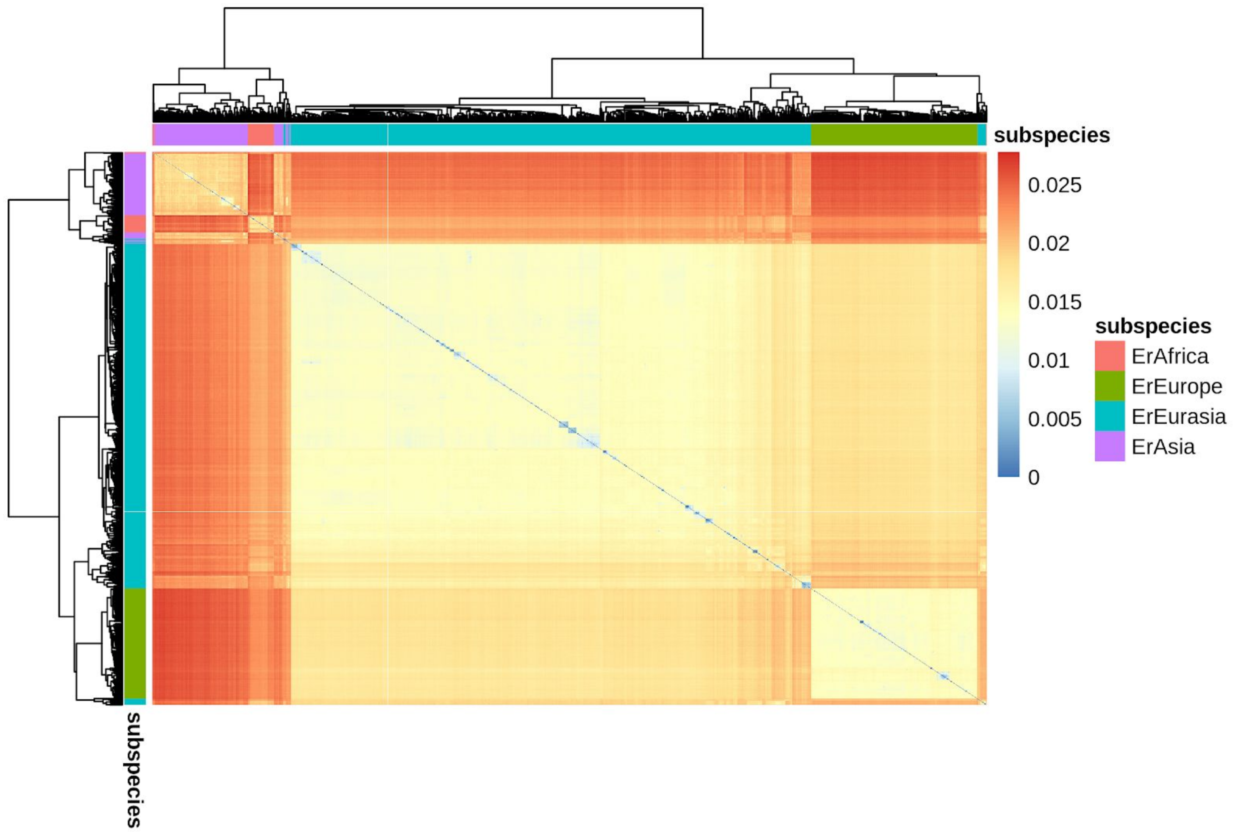

Fig S10: Heatmap of pairwise genetic distances between all high quality *E. rectale* genomes extracted from metagenomes.

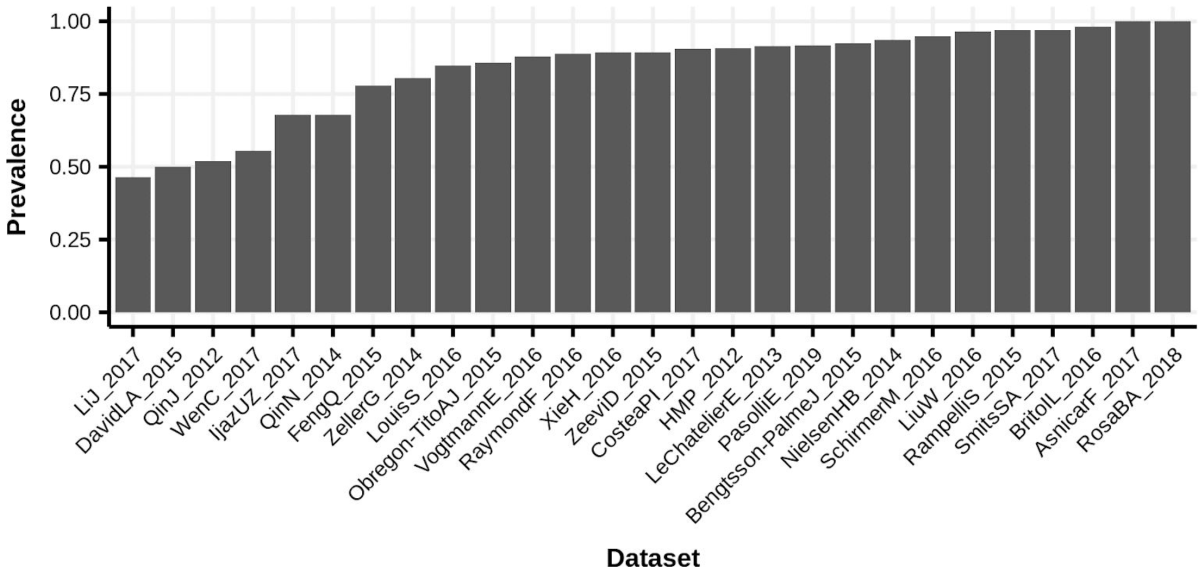

Fig S11: Prevalence proportions of *Eubacterium rectale* (Prevalence defined as relative abundance > 0.1%) in adult control samples. Some datasets did not contain any adult control samples and are thus not shown here. Relative abundances were inferred using MetaPhlAn2 [49] (**Methods**).

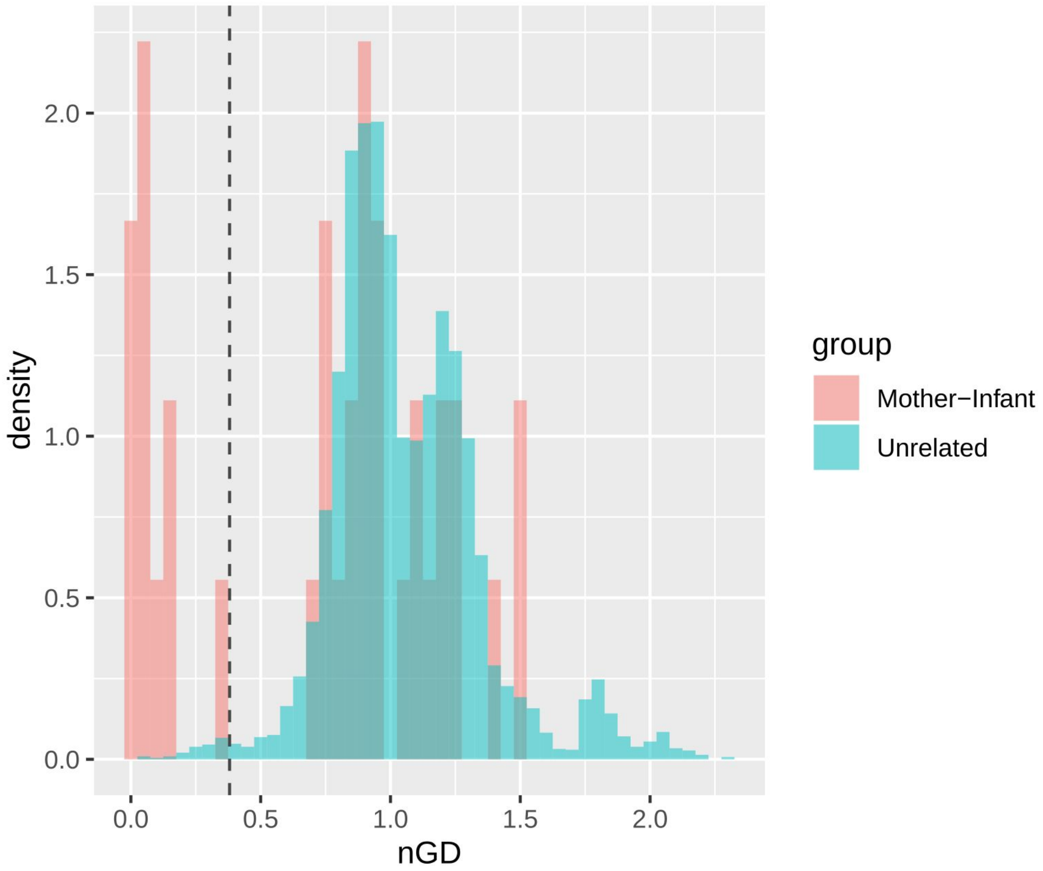

Fig S12: Distribution of genetic distances (as normalized branch lengths) between genomes isolated from Mother-infant and unrelated pairs of individuals. Datasets used were Backhed et al (N=398 samples; 96 mothers-infant pairs) [53], Asnicar et al (N=18, 5 mother-infant pairs) [54], and Ferretti et al (N=116 samples, 21 mothers and 25 infants) [55]. Dashed line indicates the 1-percentile of the distribution of unrelated individuals, used as a conservative cutoff to call a pair of strains identical (see **Methods**).

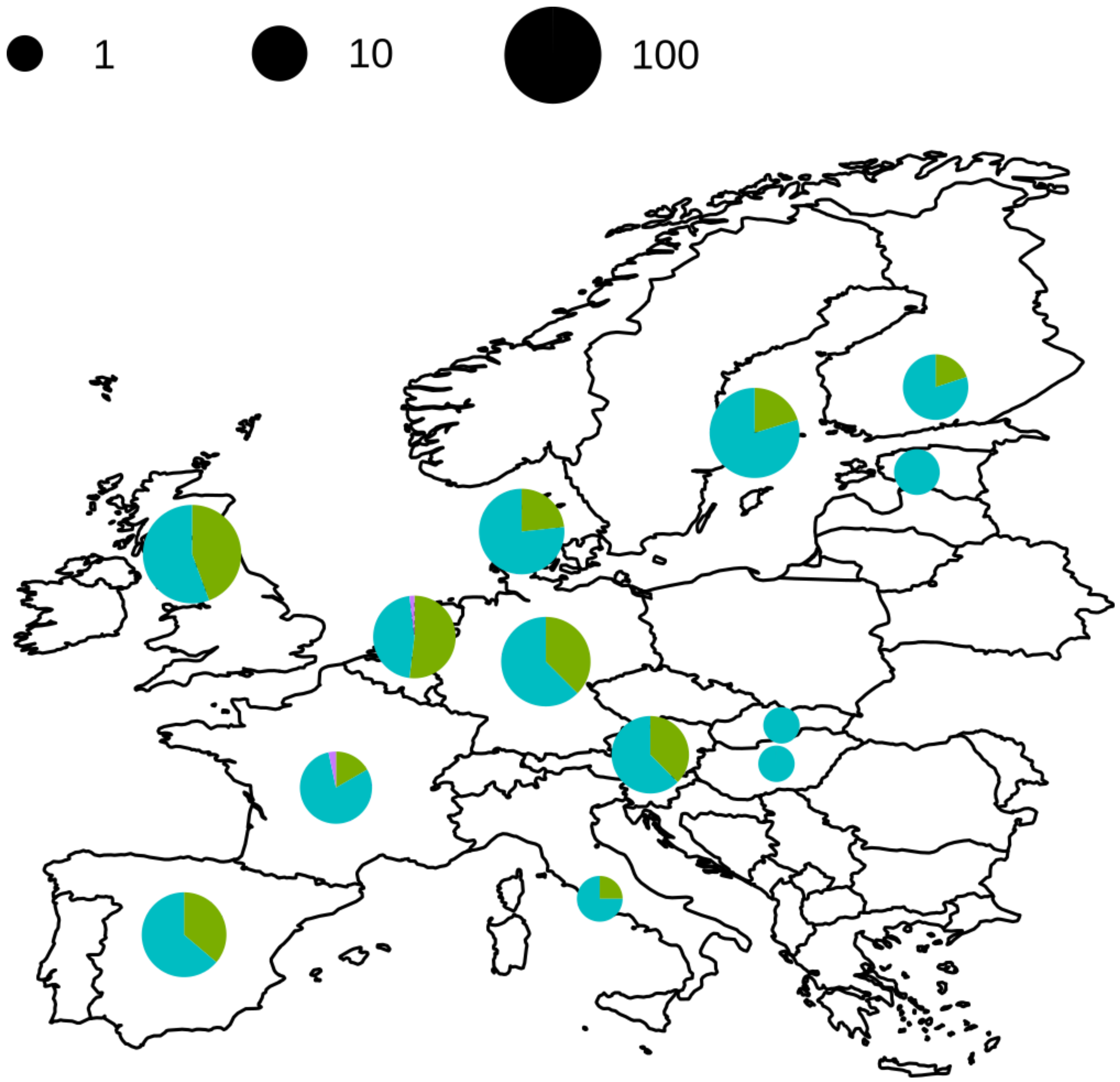

Fig S13: Ratio of subspecies prevalence within European countries. The circle size corresponds to the sample size. Cyan = ErEurasia, Green = ErEurope, Purple = ErChina.

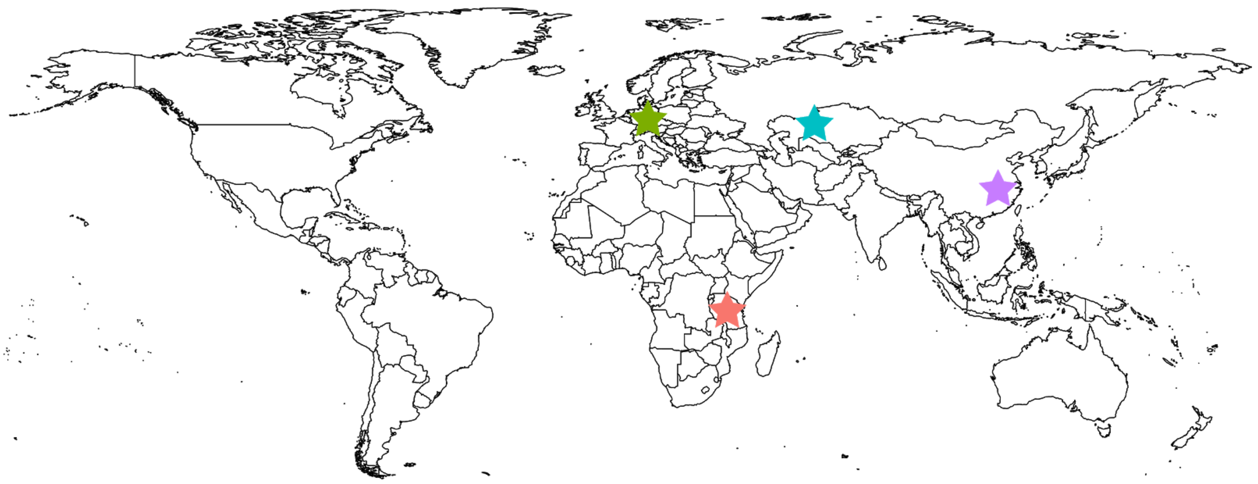

Fig S14: Point locations chosen for the four *E. rectale* subspecies. Compare with **Fig. 3**. Red = ErAfrica, Green = ErEurope, Blue = ErEurasia, Purple = ErAsia.

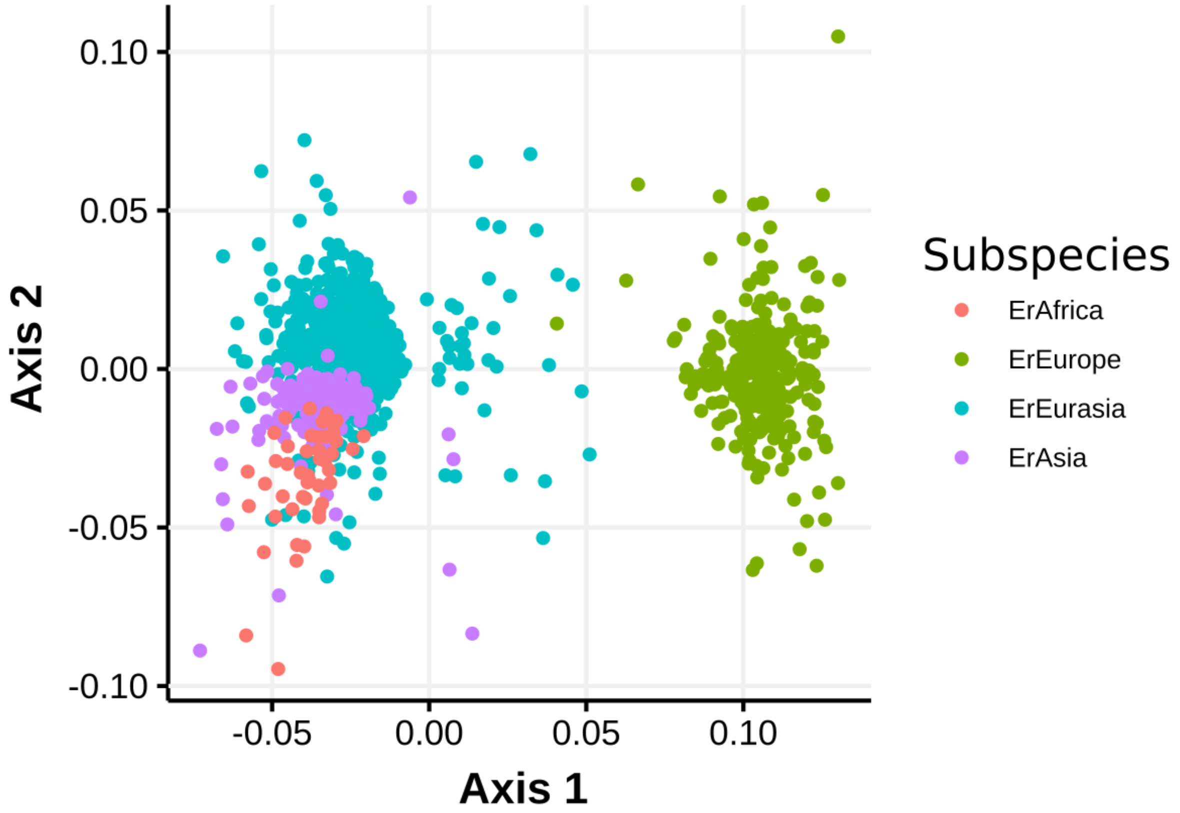

Fig S15: Ordination based on pairwise Jaccard distances computed on KO profiles (**Methods**).

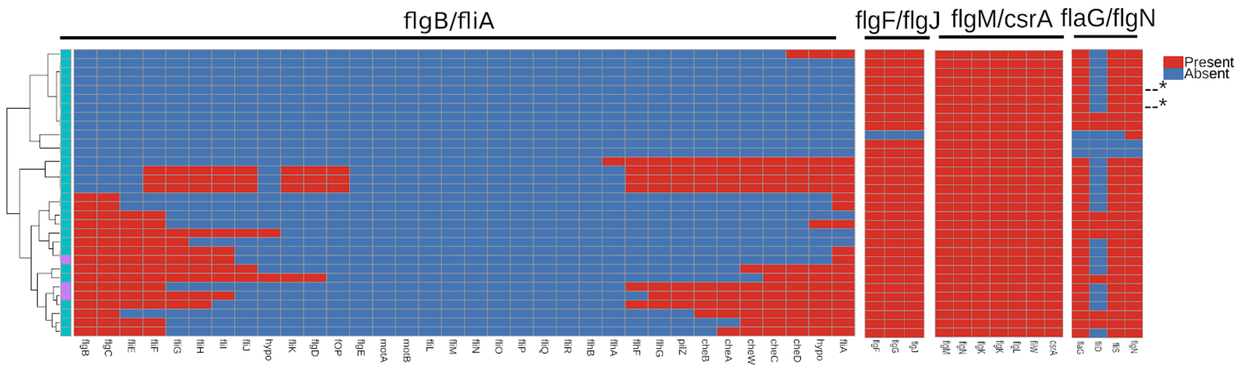

Fig S16: Heatmap of motility gene presence/absence in all those non-ErEurope strains that have some part of the *flgB*/*fliA* operon missing and that had their motility operons fully spanned on single contigs. Gene presence/absence inferred by extracting operons with bordering operon genes present (operon border genes determined using [25]). Asterisks mark isolate genomes. Row colors: Cyan = ErEurasia, Purple = ErAsia.

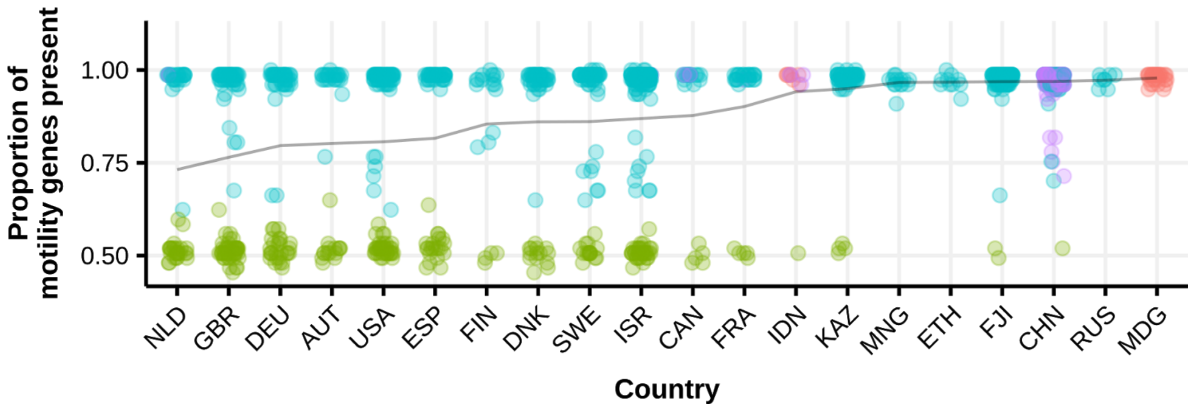

Fig S17: Proportion of motility KOs present in the HQ *E. rectale* genomes, stratified according to country and subspecies membership. Motility-association is defined as described above. Line corresponds to mean proportion per country. Only countries with at least 5 genomes are shown.

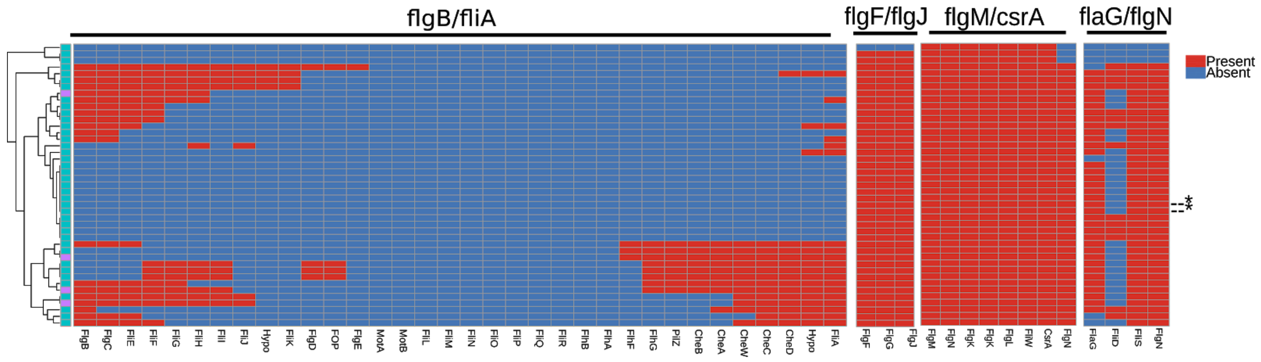

Fig S18: Heatmap of motility gene presence/absence in all those non-ErEurope strains that have some part of the *flgB*/*fliA* operon missing. Gene presence/absence inferred by mapping operon genes (sequences taken from [25]) against all extracted genomes. Asterisks mark isolate genomes. Row colors: Cyan = ErEurasia, Purple = ErAsia.

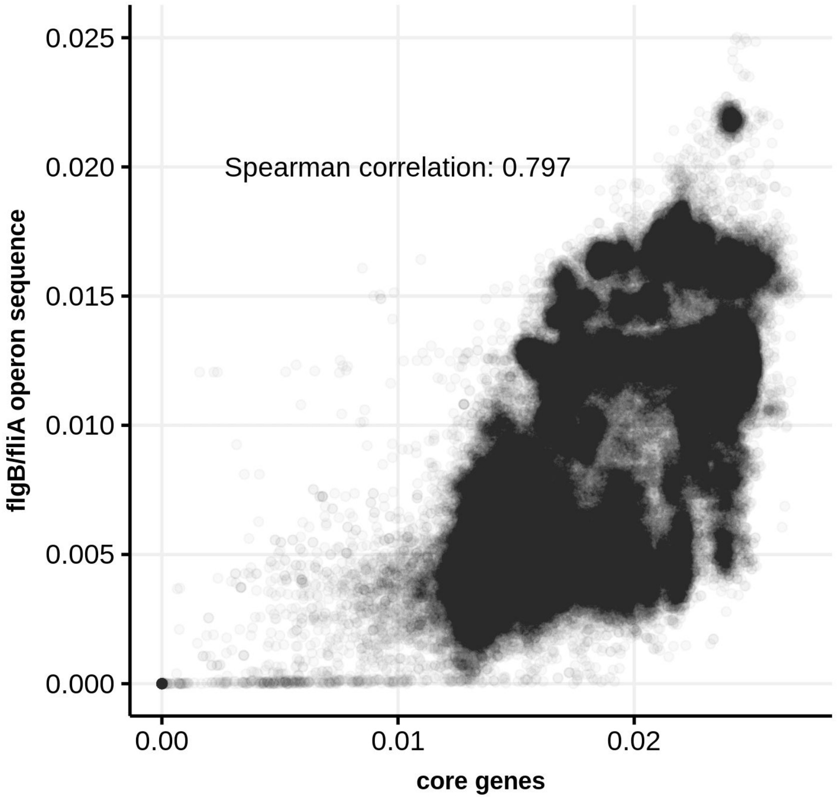

Fig S19: Scatter plot between pairwise genetic distances inferred from core genes and the largest motility operon (*flgB*/*fliA*) gene sequences for all non-ErEurope strains for which the full *flgB*/*fliA* operon could be extracted.

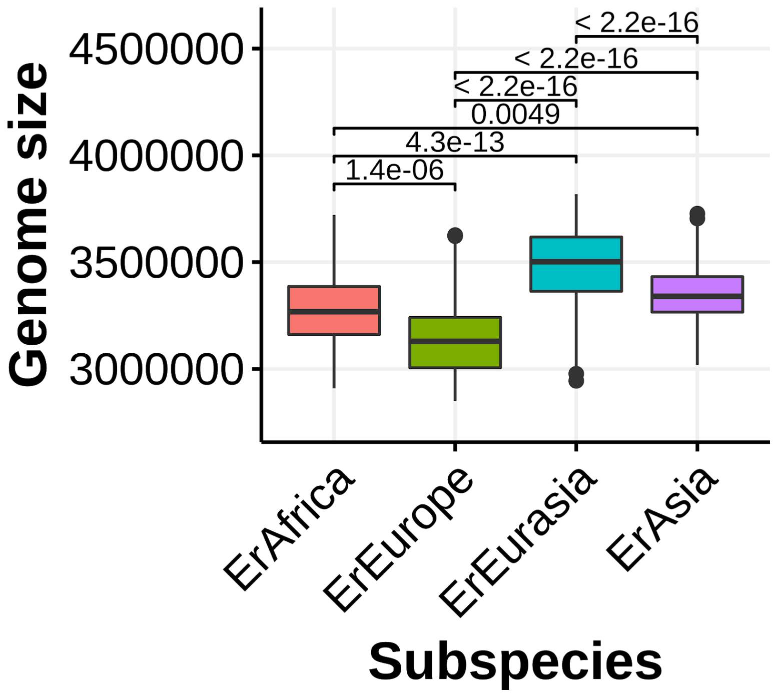

Fig S20: Boxplot of genome sizes by subspecies. P-values were calculated using a two-sided Wilcoxon test.

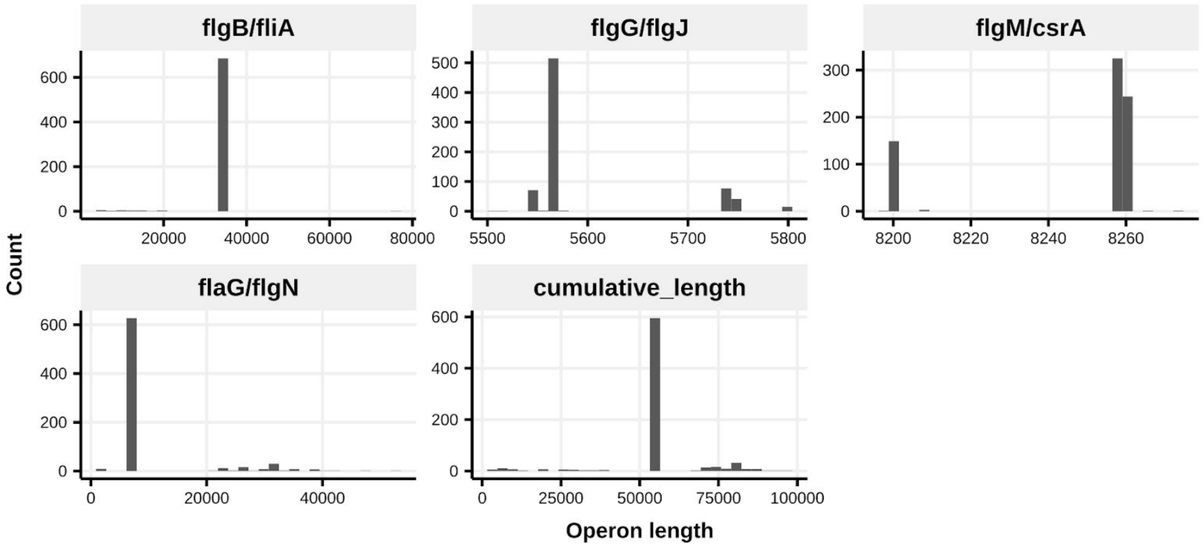

Fig S21: Histograms of operon lengths and cumulative operon length for all HQ genomes. Operon sequences and their length are generally very well conserved, with the exception of some *flaG*/*flgN* operons.

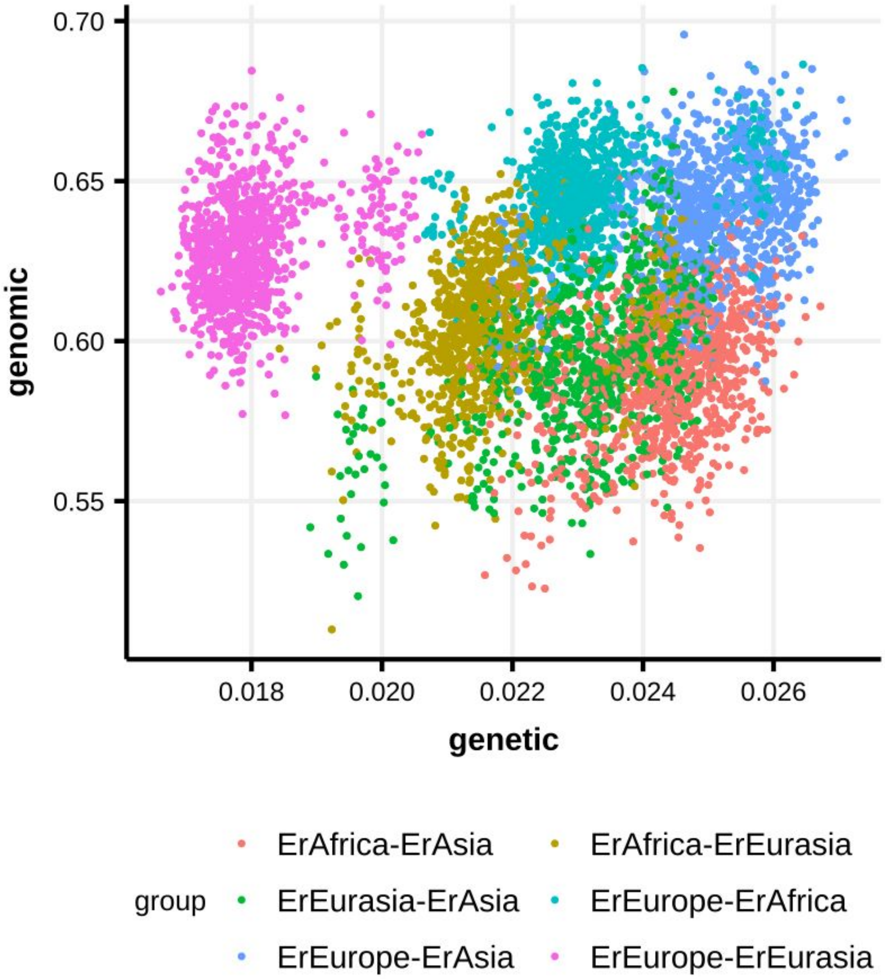

Fig S22: Scatterplot of pairwise genetic (Hamming distance on core gene alignment) and genomic (Jaccard distance on gene presence/absence, excluding gene clusters corresponding to motility operon genes) distances. For visualization purposes, genomes were subsampled to 30 random samples per subspecies.

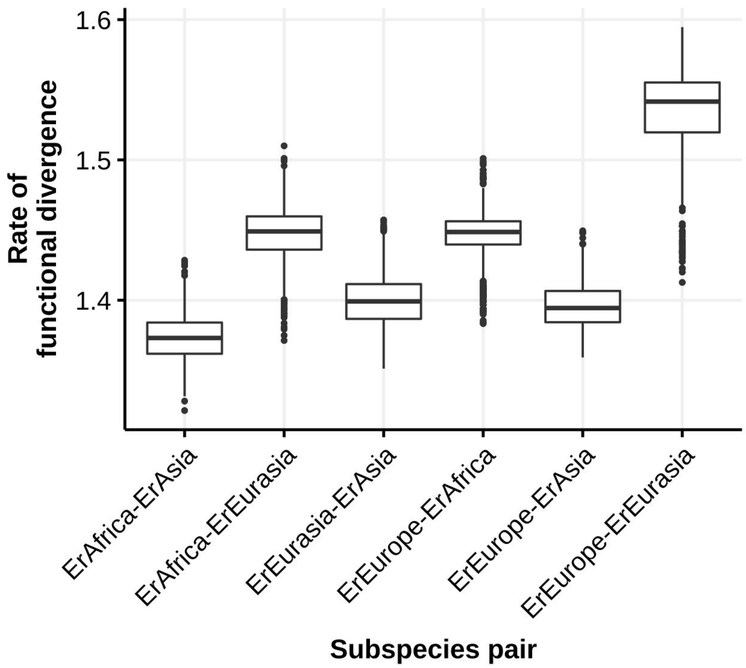

Fig S23: Functional divergence rates of pairs of subspecies, calculated by dividing pairwise inter-subspecies genomic distances by their corresponding genetic distance (**Methods**).

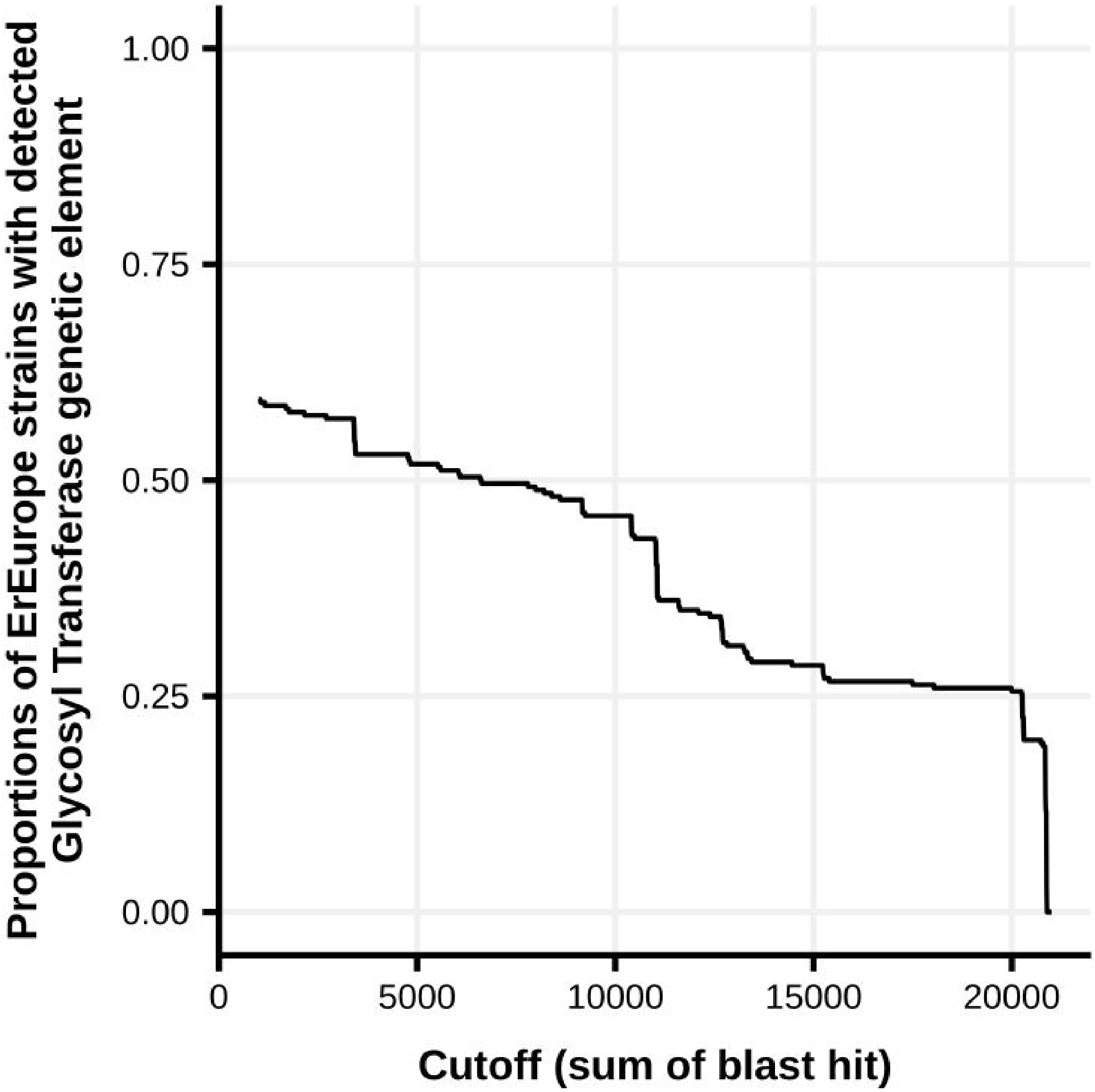

Fig S24. Line plot showing the proportion of detected GT-enriched genomic islands as a function of the total length of blast hits. This plot suggests that the true fraction of ErEurope strains possessing the genetic element is distinctly higher than ~21% (corresponding to the proportion of ErEurope strains where the full genetic element could be detected), probably due to partial assembly.

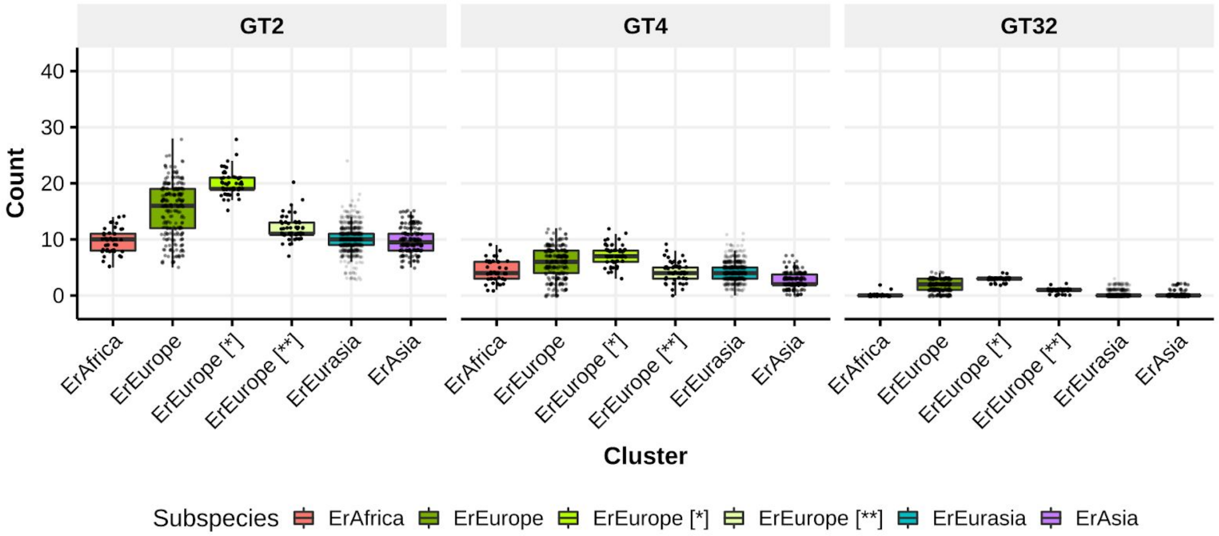

Fig S25: Boxplots of genome-wide GT counts stratified by subspecies. [*] corresponds to counts for those ErEurope strains with completely extracted GT-enriched genomic islands, [**] corresponds to those ErEurope but with counts corresponding to the GT-enriched genomics island removed. See **Fig. 6A**.

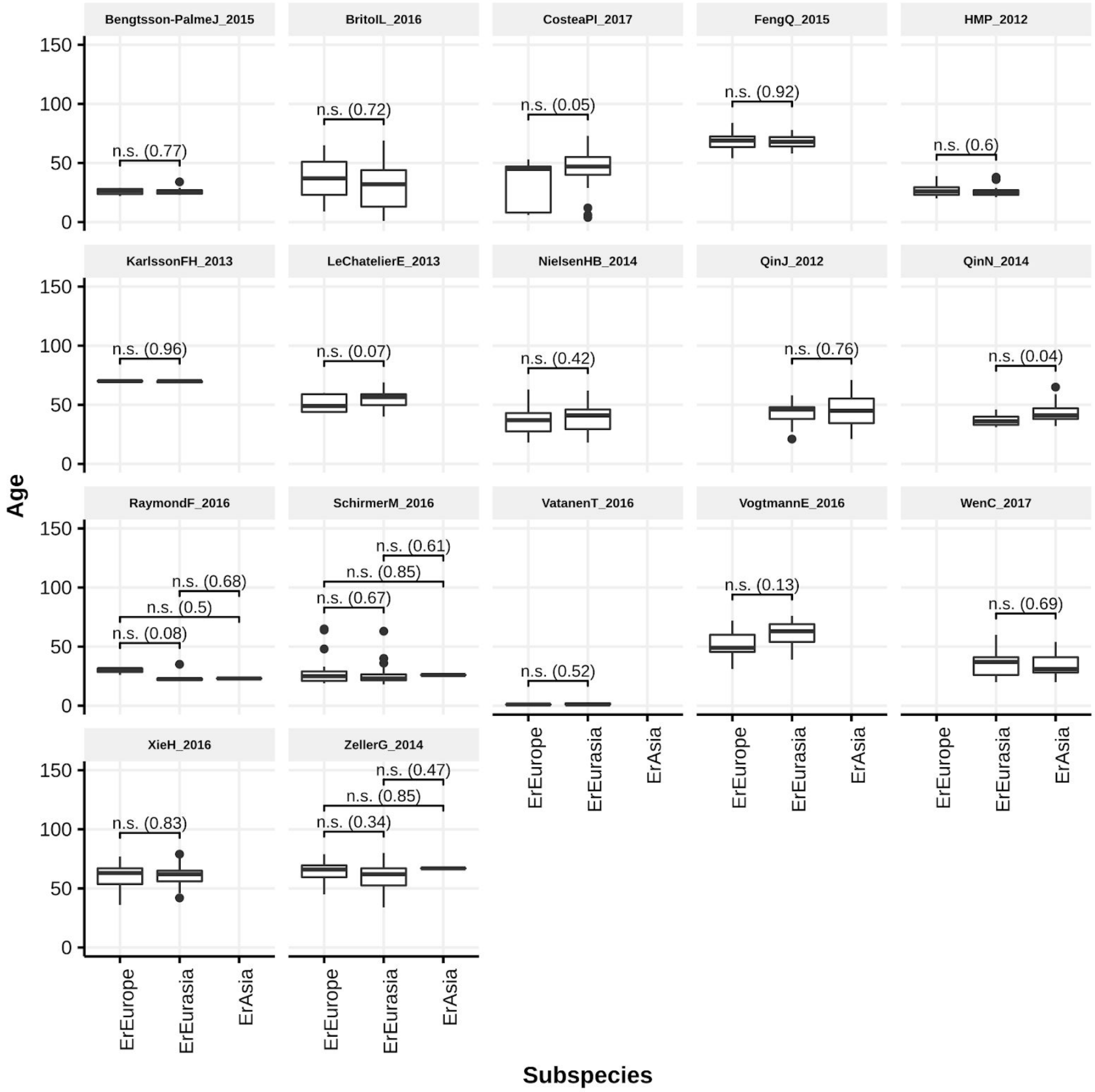

Fig S26: Boxplots of age grouped by subspecies. Label corresponds to significance level at 5% FDR (FDR-correction using Benjamini-Hochberg), numbers in parenthesis correspond to uncorrected p-values. P-values calculated using two-sided Wilcoxon tests.

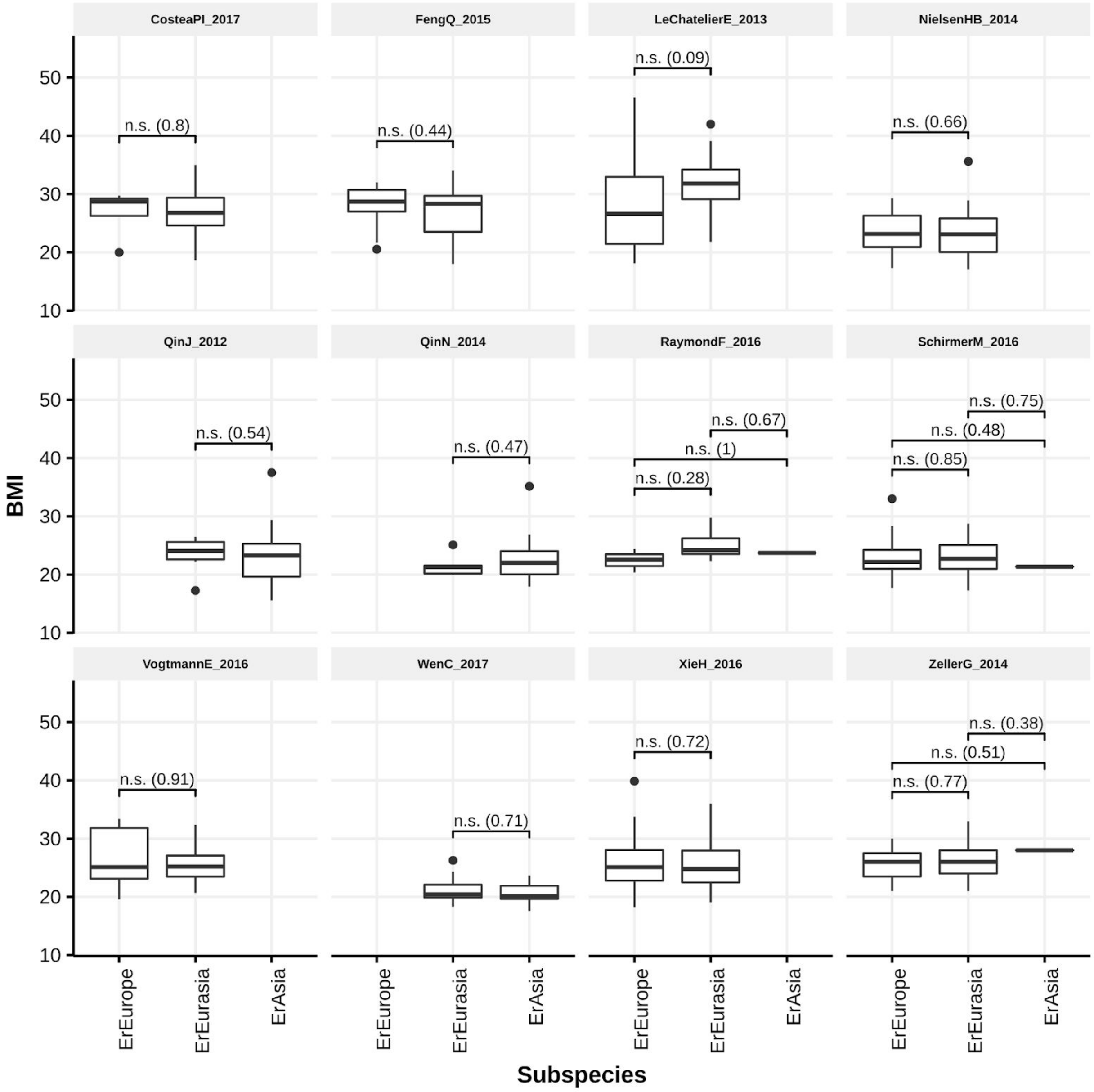

Fig S27: Boxplots of BMI grouped by subspecies. Label corresponds to significance level at 5% FDR (FDR-correction using Benjamini-Hochberg), numbers in parenthesis correspond to uncorrected p-values. P-values calculated using two-sided Wilcoxon tests.

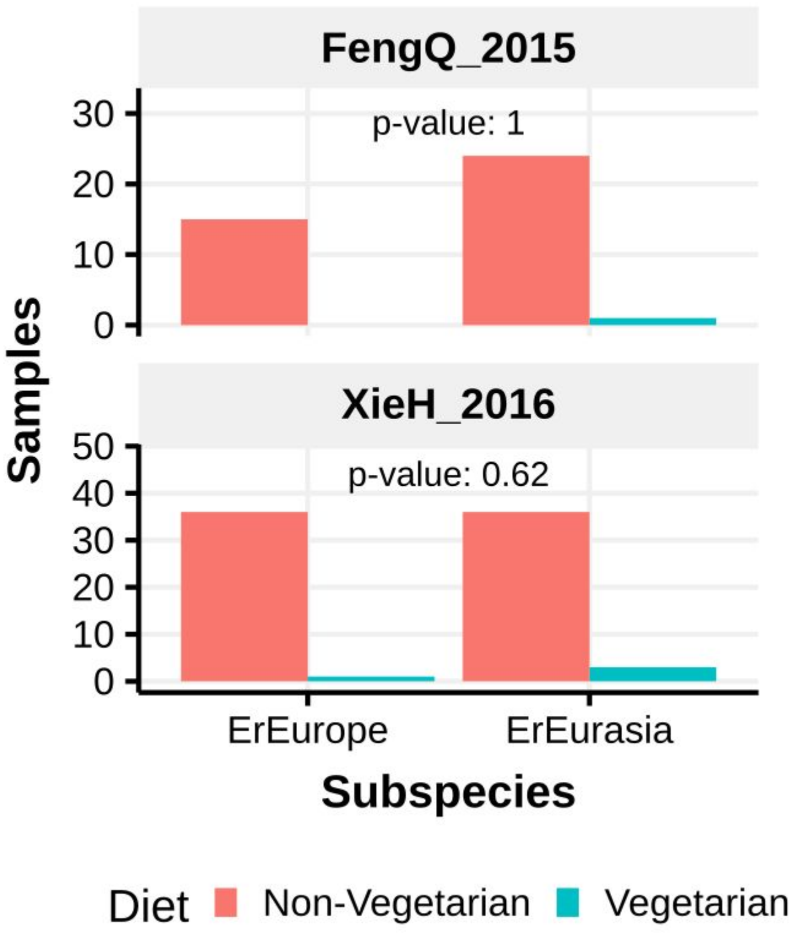

Fig S28: Bar Plots showing the distribution of ErEurope and ErEurasia in two datasets where qualitative diet information (vegetarian/non-vegetarian) was available. P-values were calculated using a two-sided Fisher test.

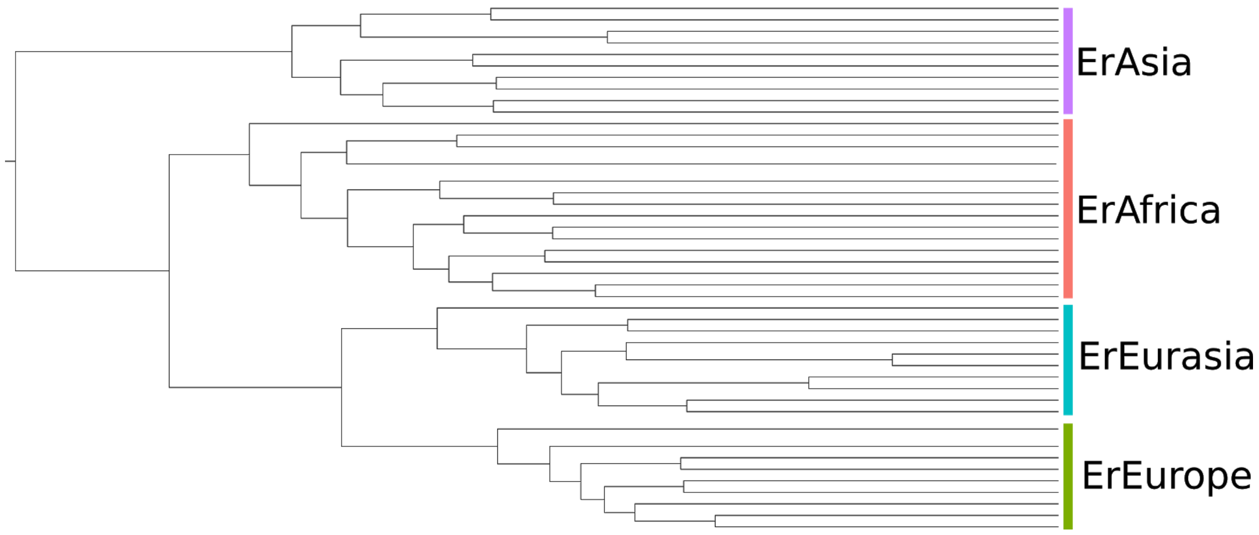

Fig S29: Rooted bayesian phylogeny [78] built on a randomly chosen, representative subset of samples per subspecies (**Methods**). Clade topology is consistent across subsamples.

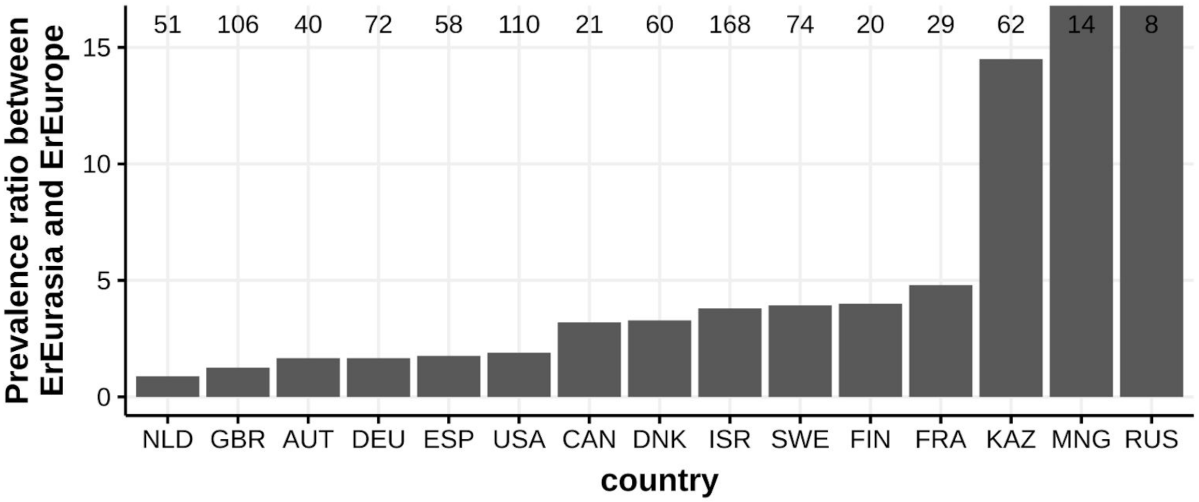

Fig S30: Ratios of prevalence between ErEurasia and ErEurope for Eurasian/North American countries. Mongolia and Russia have undefined ratios, since no ErEurope genomes were reconstructed from samples originating in these countries. The number above the bars indicates the number of genomes reconstructed from each country. Only countries with at least 5 genomes are shown.

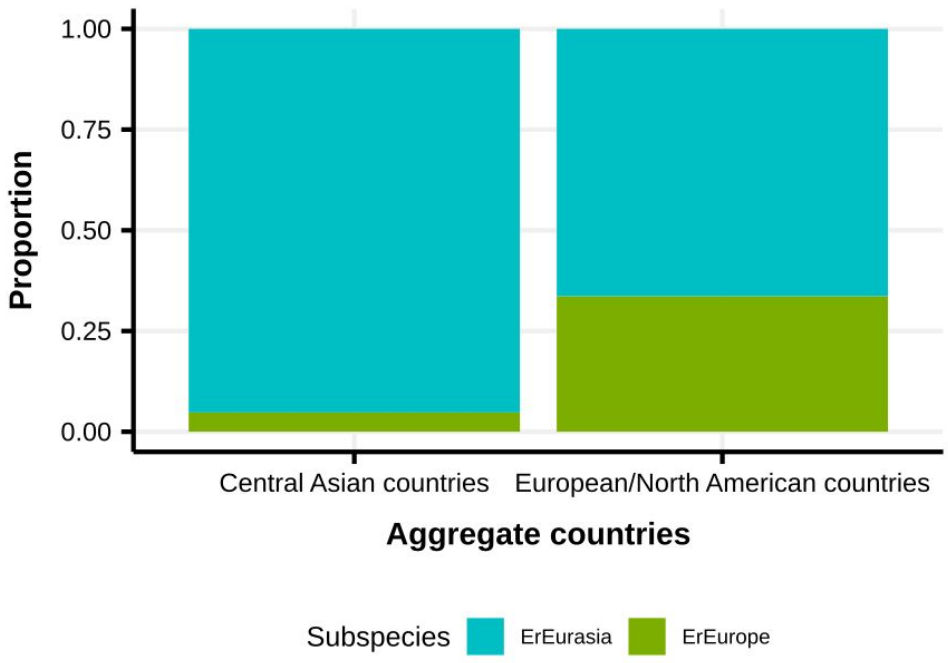

Fig S31: Prevalence ratios of ErEurasia and ErEurope between Kazakhstan, Mongolia and Russia (in aggregate) against the remaining countries in Europe and North America. P-value for differential prevalence is 9.8E-09 (Fisher test).
